# Food Consumption Among the Adult Men and Women of Bangladesh and Its Adherence to National Food‐Based Dietary Guidelines

**DOI:** 10.1002/fsn3.71056

**Published:** 2025-10-08

**Authors:** Md. Hafizul Islam, Oumma Halima, Nazma Shaheen

**Affiliations:** ^1^ Institute of Nutrition and Food Science University of Dhaka Dhaka Bangladesh

**Keywords:** adherence to dietary guidelines, Bangladesh, dietary gap, food consumption, food‐based dietary guidelines

## Abstract

The present study aimed to identify the food and nutrient intake among the adult population of Bangladesh and its adherence to Bangladesh's food‐based dietary guidelines (FBDG). The consumption data of 1551 adult men, non‐pregnant and non‐lactating (NPNL), and pregnant and lactating women (PLW) were obtained from the Nutrition Survey of Bangladesh (NSB) 2017–18. The NSB survey collected the participants' individual‐level intake using the food weighed record method. The usual intake of different food groups was compared with the FBDG of Bangladesh to assess adherence to the recommendation. Their dietary macronutrient intake was compared with the age‐ and sex‐specific acceptable macronutrient distribution range. The probability of micronutrient adequacy was calculated considering the age‐, sex‐, and physiological state‐specific Estimated Average Requirements. Kruskal–Wallis and chi‐squared tests were used to compare differences among men, NPNL, and PLW in nutrient adequacy and adherence to FBDG. Adherence to dietary guidelines was low, especially for fruits (4.1%), milk (5%), and vegetables (6%–7%). Women (NPNL and PLW) had higher adherence to cereals, while men showed greater adherence to fruits, animal‐source foods, and milk. Almost all the participants met the protein intake guideline. In contrast, most participants consumed fat below and carbohydrates above the recommended range, with similar adherence across men, NPNL, and PLW and no significant differences. Calcium, riboflavin, vitamin B12, and vitamin C adequacy were zero among the participants across all groups. Niacin was adequate (100%), while other micronutrients showed low adequacy. Men had higher iron and thiamin adequacy; women, especially PLW, had better magnesium, zinc, folate, and vitamin B6. MPA was highest in men (26.3%), followed by NPNL (22.2%) and PLW (21.7%), with significant sex differences. Thus, the intake of the above‐mentioned food groups should be increased to meet their dietary recommendations and ensure the adequacy of different micronutrients.

## Graphical abstract

Among Bangladeshi adults, diets were dominated by cereals and grains, with low intake of dairy, fruits, vegetables, pulses, and animal‐source foods, leading to poor adherence to national dietary guidelines. Energy came largely from carbohydrates, fat intake was insufficient, and although protein intake was mostly adequate, it was primarily plant‐based. Many, especially women, had inadequate intakes of key micronutrients like riboflavin, calcium, and vitamin A, highlighting the need to increase consumption of under‐consumed food groups to improve nutrient adequacy.

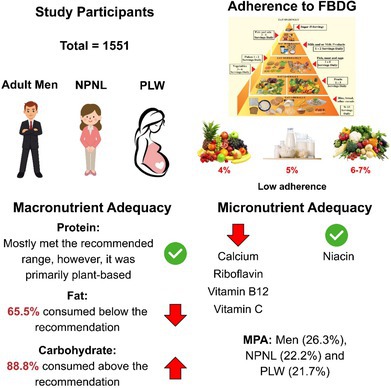

## 1 Introduction

The dietary habits and lifestyle of the people of Bangladesh have been evolving over the years. Different energy‐dense and ready‐to‐eat food items have been introduced into their diets, especially in urban areas (Banik et al. 2020; Choudhury et al. 2021; Goon 2014). In addition, they have less involvement in physical activity than they did earlier, which is one of the major contributors to their transition to obesity from normal body weight (Al Muktadir et al. 2019; Islam et al. 2020). Moreover, unhealthy food choices, a sedentary lifestyle, and uncontrolled body weight might result in various non‐communicable diseases (Riaz et al. 2020; Sharma et al. 2023). However, evidence suggests that cereal‐based foods still dominate the diets of the Bangladeshi population and that there are inadequate diverse food items (Arsenault et al. 2013; Islam, Jubayer, et al. 2023). This inadequate dietary diversity and plant‐based diets lead to an insufficient intake of critical micronutrients. Among the reproductive‐aged women of Bangladesh, only 40% had adequate dietary diversity, with especially poor intake of dairy products and fruits. In contrast, many of them had inadequate vitamin A, riboflavin, folate, calcium, iron, zinc, and so forth (Akter et al. 2021; Arsenault et al. 2013; Islam, Jubayer, et al. 2023; Islam et al. 2022).

The Bangladesh food‐based dietary guidelines were first published in 2013 to promote normative care and prevent nutritional deficiency disorders in the Bangladeshi population (BIRDEM 2014). The guideline was amended again in 2020 based on detailed national survey data and other neighboring nations' dietary guidelines. These guidelines focused on food groups rather than nutrients, partly to facilitate easier communication and implementation by the general population. These measures encourage the intake of recommended cereals, fruits, vegetables, legumes, animal‐source foods, nuts and seeds, and milk and milk products while limiting the intake of sugar, sugar‐sweetened beverages, fats and oils, oily foods, and salt. In addition, the guidelines recommend following the ICMR‐NIN prescribed age‐ and sex‐specific intake level of energy, macronutrients, and micronutrients (BIRDEM 2014; ICMR‐NIN 2020).

In addition to advancement, turning these thoughts into action is a challenging task that necessitates enabling components at both the personal and environmental levels. As a result, evidence‐based information on the existing gaps between the guidelines and the population's dietary and lifestyle practices, as well as demographic and socioeconomic factors influencing adherence to these guidelines within the country, is required to promote and implement dietary guidelines. Furthermore, effective implementation of dietary guidelines in any given community would require measuring adherence, and understanding adherence to these rules may minimize the risk of chronic diseases and all forms of malnutrition. Different countries, including Egypt (Ansari and Samara 2018), Qatar (Thani et al. 2018), Germany (Stroebele‐benschop et al. 2018), Gambia (Ali et al. 2022), Australia (Gopinath et al. 2014; Russell et al. 2013), and the Netherlands (Braver et al. 2020), have evaluated the dietary intake of the population with their national and international dietary guidelines. Even though a few studies in Bangladesh explored the dietary intake of the population of different ages (Akter et al. 2021; Arsenault et al. 2013; Islam, Jubayer, et al. 2023; Islam et al. 2022), there have been no studies or investigations that characterize the population's adherence to the developed food‐based dietary guidelines of Bangladesh. However, to improve the population's nutrition situation, it is fundamental to evaluate their dietary habits against the recommended dietary guidelines. Identifying the population at risk of dietary inadequacy through examining their adherence to FBDG will raise concerns about taking necessary intervention policies. The Institute of Nutrition and Food Science, University of Dhaka, conducted the Nutrition Survey of Bangladesh (NSB) in 2017–18 (Shaheen et al. 2021). The survey included the latest nationally representative individual intake data of the Bangladeshi population (FAO/WHO 2023). Based on the dietary data of the NSB survey, the present study aims to examine the food and nutrient intake among the adult men and women of Bangladesh and evaluate their adherence to the national food‐based dietary guidelines.

## 2 Methods

### 2.1 Study Design and Source of Data

The study was designed to find the adherence of the dietary intake of adult men and women of Bangladesh to the national food‐based dietary guidelines. The consumption data were obtained from the Nutrition Survey of Bangladesh (NSB) 2017–18 (FAO/WHO 2023). The survey was conducted by the Institute of Nutrition and Food Science (INFS), University of Dhaka. The NSB survey included dietary consumption data of 3457 individuals of different age and sex groups. From these consumption data, 1551 adult men and women (19–50 years) were included in the current study. The women comprised both women of reproductive age, pregnant, and lactating women. There was no data collection throughout the fasting month of Ramadan and 2 weeks after the day of the Muslim holy festival of livestock sacrifice. Between April 2017 and March 2018, the survey covered the whole crop and weather cycle (Shaheen et al. 2021).

### 2.2 Dietary Intake Assessment

The NSB survey collected individual‐level intake of the household members using the weighed food record method. Household utensils and weighing scales were used to measure the cooked weight of the food items consumed by the individual. The cooked weight of the ingredients was converted to raw weight using a conversion factor. The Bangladeshi Food Composition Table (FCT) (Shaheen et al. 2014) was used to estimate the nutrient content of the food items that adult men and women consumed. The survey collected repeated consumption data from 10% of the samples. Using the repeated consumption data, usual dietary intake was calculated to capture intra‐individual variability.

### 2.3 Assessing Adherence to the National Food‐Based Dietary Guidelines

Based on the dietary guidelines of Bangladesh (BIRDEM 2014), the dietary intake of adult men and women was classified into 10 different food groups along with salt, as highlighted in Table 1. The amount of food consumed from these food groups was compared with the dietary guidelines of Bangladesh for the adult population. To determine adherence to the guideline, the intake level was evaluated against the lower value of the recommended range for leafy vegetables (125 g/day), non‐leafy vegetables (300 g/day), fruits (80 g/day), fish, meat, and egg (100 g/day), pulse and legumes (30 g/day), and milk and milk products (150 g/day) (Table 1). On the other hand, the respondents were considered to have compliance with the guidelines when their intake of cereals, roots, and tubers (270–450 g/day) and oils (15–30 g/day) was found to be within the recommended range. In contrast, sugar (≤ 25 g/day) and salt (≤ 5 g/day) intake was found to be below the upper level.

**TABLE 1:** Food groups and recommendations of FBDG of Bangladesh.

Food group	Description	Serving size (g)	Number of servings
Cereals, roots, and tubers	This group is also called starchy staples. This group includes all types of seeds or grains from cereals such as rice, maize, wheat, barley, sorghum, pasta, and so forth. Roots and tubers include potatoes, white‐fleshed sweet potatoes, plantains, and so forth	30	9–15
Leafy vegetables	This group includes leafy vegetables such as amaranth, spinach, lettuce, and so forth	125	1–2
Non‐leafy vegetables	This group includes non‐leafy vegetables such as cauliflower, green beans, okra, tomatoes, cucumber, lettuce, and so forth	150	2–4
Fruits	Fresh fruits such as banana, mango, guava, hog plum, jackfruit, orange, and so forth. Fruit juices and dried and canned fruits are also included in this group	80	1–3
Fish, meat, and egg	This group includes food from animal sources such as red meat, chicken, fish, shellfish, sausages, and so forth	100	1–4
Pulse and legumes	Peas, beans, and lentils, which grow as seeds inside a pod, are collectively known as legumes or pulses. This group includes members of the plant family Leguminosae, such as beans, peas, lentils, soybeans, and so forth	30	1–2
Milk and milk products	This group includes milk, yogurt, cheese, salt, yogurt, and so forth	150	1–2
Fats and oils	Edible oils used in cooking include soybean oil, palm oil, groundnut oil, and mustard oil, butter, ghee	5	3–6
Sugar	This group includes added sugar in sweets and desserts: Sugar, jam and honey, ice cream, pudding, custard, chocolate, cake, and so forth	5	≤ 5
Salt	Iodized salt	5	< 1

### 2.4 Assessing the Adequacy of Dietary Nutrient Intake

The adequacy of macronutrient intake was determined by comparing the distribution of percent dietary energy of protein, carbohydrate, and fat with the acceptable macronutrient distribution range (AMDR) of the Indian Council of Medical Research (ICMR) (ICMR‐NIN 2024). The guidelines recommended that 7.5%–20%, 20%–30%, and 50%–60% of daily energy should come from protein, fat, and carbohydrate, respectively. When the intake of different macronutrients was found within the recommended range, they were considered to have adequate intake of that macronutrient. On the other hand, to evaluate micronutrient adequacy in the diet of adult men and women of Bangladesh, the probability of nutrient adequacy was calculated. A total of 12 micronutrients were considered to assess the probability of adequacy. To assess probability adequacy, age‐, sex‐, and physiological state‐specific Estimated Average Requirement (EAR) of micronutrients recommended by the ICMR was used (ICMR‐NIN 2020, 2024). In the absence of such national guidelines, we used the ICMR‐NIN recommendations as a proxy, as there is geographical proximity, cultural similarities, and comparable dietary patterns between India and Bangladesh.

### 2.5 Statistical Analysis

Individual‐ and household‐level background characteristics and adherence to dietary recommendations were reported as frequencies and percentages. The normality of the distributions of continuous variables (probability of nutrient adequacy) was assessed through the Kolmogorov–Smirnov test and Shapiro–Wilk test. The probability of nutrient adequacy was not normally distributed and reported as median. To compare differences among men, NPNL, and PLW in probability of nutrient adequacy, the Kruskal–Wallis test was used, followed by group comparisons. Differences were considered significant at *p* < 0.05. Moreover, a chi‐squared test was used to find the gender‐based differences in adherence to dietary guidelines and nutrient adequacy. All the statistical analyses were conducted using the SPSS software version 25.0.

## 3 Results

### 3.1 Sociodemographic Characteristics of the Participants

The background characteristics of the study participants have been presented in Table S1. The mean (SD) age of the participants was 30.91 (7.88) years, and about 86% were between 19 and 40 years. The number of men and women participants was almost equal. About two‐thirds of them (64.3%) were from rural areas. About 37.3% had secondary, and 12.6% had higher secondary/above education, while 20% of the participants had no formal education. Among the women, a little over half of the women (58.5%) are non‐pregnant and non‐lactating (NPNL), whereas 41.5% are either pregnant or lactating (PLW).

### 3.2 Adherence to the Food‐Based Dietary Guidelines

Overall adherence to the food‐based dietary guidelines was low for several key food groups, including fruits, milk and milk products, and vegetables (Table 2). Specifically, fruit consumption was low across all groups, with only 4.1% adherence overall. Milk products and vegetables also had low adherence rates, around 5% and 6%–7%, respectively. Significant differences between men, NPNL, and PLW were observed for several foods. NPNL (39.9%) and PLW (35.9%) had higher adherence to cereals, roots, and tubers compared to men (16.2%). Men showed greater adherence to fruits (5.8%), meat, fish, eggs (48.3%), and milk products (8.4%) than women. Non‐leafy vegetable consumption was also higher among men (7.9%), though overall vegetable intake remained low. Salt adherence was higher among women, with 54.1% of NPNL and 56.5% of PLW adhering compared to 45.1% of men. Adherence to fat consumption was reported as significantly high in women (around 40%) compared to men (32%). In contrast, adherence to sugar intake was uniformly high across groups, with no significant differences.

**TABLE 2:** Adherence of different food group consumption to the food‐based dietary guidelines of Bangladesh.

Food groups	Overall *n* (%)	Men *n* (%)	NPNL *n* (%)	PLW *n* (%)	*p*
Cereals, roots, and tubers	426 (27.5)	123 (16.2)	185 (39.9)	118 (35.9)	< 0.001
Non‐leafy vegetables	98 (6.3)	60 (7.9)	19 (4.1)	19 (5.8)	0.026
Leafy vegetables	115 (7.4)	56 (7.4)	36 (7.8)	23 (7.0)	0.920
Pulses and legumes	348 (22.4)	176 (23.2)	104 (22.4)	68 (20.7)	0.651
Fruits	64 (4.1)	44 (5.8)	14 (3.0)	6 (1.8)	0.004
Meat, fish, and egg	668 (43.1)	366 (48.3)	181 (39.0)	121 (36.8)	< 0.001
Milk and milk products	82 (5.3)	64 (8.4)	9 (1.9)	9 (3.9)	0.947
Fats	561 (36.2)	245 (32.3)	182 (39.2)	134 (40.7)	0.008
Sugar	1515 (97.7)	737 (97.2)	453 (97.6)	325 (98.8)	0.293
Salt	779 (50.2)	342 (45.1)	251 (54.1)	186 (56.5)	0.001

*Note:* *p*‐values from chi‐squared test.

Abbreviations: NPNL, non‐pregnant and non‐lactating; PLW, pregnant and lactating.

### 3.3 Adherence to Recommended Macronutrient Intake

**FIGURE 1:**
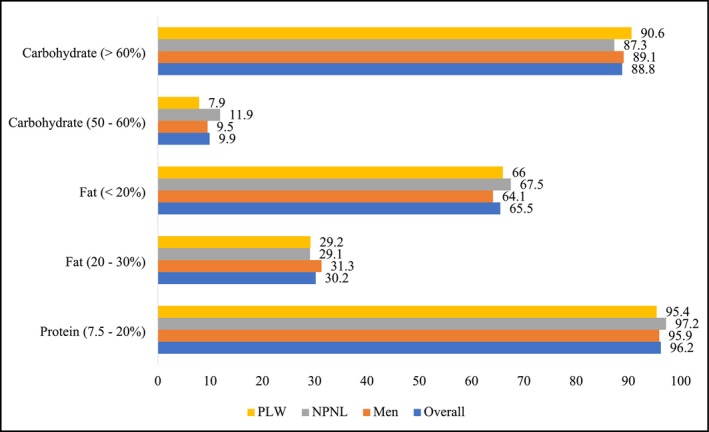
Adherence of the study participants to adult macronutrient distribution ranges (AMDR).

Adherence to recommended macronutrient distribution ranges (AMDR) among men, NPNL, and PLW is presented in Figure 1. Overall, 96.2% of participants met the protein intake guideline (7.5%–20% of total energy), 30.2% met the fat intake guideline (20%–30% of total energy), while only 9.9% met the carbohydrate intake guideline (50%–60% of total energy). However, the majority of the participants (65.5%) consumed below the recommended range of fat (< 20% of total energy). On the contrary, the consumption of carbohydrate exceeded the upper recommended range (> 60% of total energy) for a higher portion of the participants. The adherence rates were similar across the groups for all three macronutrients, with no statistically significant differences observed: protein (*p* = 0.376), fat (*p* = 0.657), and carbohydrate (*p* = 0.165). This indicates consistent compliance with macronutrient distribution recommendations among men, NPNL, and PLW.

### 3.4 Contribution of Different Food Groups to Daily Energy and Macronutrients

The contribution of different food groups to daily energy and macronutrient intake reveals a heavy dependence on a few key sources (Table 3). Cereals, roots, and tubers are the dominant contributors, providing 72.2% of total energy, 92.7% of carbohydrates, 55.1% of protein, and 7.0% of fat, indicating their central role in the diet. Meat, fish, and eggs contribute significantly to protein intake (29.6%) and also provide 12.7% of dietary fat, despite their low contribution to energy (5.8%). Pulses and legumes contribute to 6.4% of protein and 2.2% of energy, while vegetables and fruits offer minimal contributions across all macronutrients. On the other hand, fats and oils provide a substantial 13.7% of energy and 74.2% of total fat. Milk and milk products make only minor contributions to all nutrients.

**TABLE 3:** Contribution of different food groups to daily energy, protein, carbohydrate, and fat.

Food groups	Energy	Protein	Carbohydrate	Fat
Cereals, roots, and tubers	72.2	55.1	92.7	7.0
Pulses and legumes	2.2	6.4	1.9	0.5
Non‐leafy vegetables	2.4	4.2	2.4	0.5
Leafy vegetables	0.3	1.0	0.1	0.2
Fruits	0.5	0.3	0.5	0.2
Meat, fish, and egg	5.8	29.6	0.0	12.7
Milk and milk products	1.1	2.0	0.6	3.0
Fats	13.7	0.0	0.0	74.2
Others	1.7	1.4	1.7	1.5

### 3.5 Probability of Micronutrient Adequacy

The median probability of micronutrient adequacy (PA) among men, NPNL, and PLW reveals widespread inadequacy across all groups (Table 4). Notably, calcium, riboflavin, vitamin B12, and vitamin C adequacy were zero in most of the population and across all subgroups, indicating severe deficiencies. While niacin adequacy was universally sufficient (100%), other micronutrients like zinc, folate, and vitamin A showed very low adequacy across all groups. Men had significantly higher adequacy for iron (34.8%) and thiamin (40.7%) compared to NPNL and PLW. In contrast, women, especially PLW, had better adequacy for magnesium, zinc, folate, and vitamin C. For instance, folate adequacy was 28.9% in PLW versus only 1.9% in men. Vitamin B6 adequacy was higher in women (37%–39%) than in men (10.9%). The overall mean probability of adequacy (MPA) was highest in men (26.3%), followed by NPNL (22.2%) and PLW (21.7%), with significant differences observed between men and women.

**TABLE 4:** Probability of micronutrient adequacy among the study participants.

Micronutrients	Overall	Men	NPNL	PLW
Calcium	0	0^a^	0^b^	0^b^
Magnesium	14.81	12.82^b^	17.33^a^	14.35^a^
Iron	21.98	34.80^a^	15.65^b^	14.96^b^
Zinc	0.78	0.39^b^	1.57^a^	1.18^a^
Thiamin	16.48	40.7^a^	14.31^b^	8.93^c^
Riboflavin	0	0^a^	0^b^	0^b^
Niacin	100	100^b^	100^a^	100^a^
Vitamin B6	36.63	10.89^a^	36.84^a^	38.58^a^
Folate	9.13	1.91^b^	19.35^a^	28.93^a^
Vitamin B12	0	0^a^	0^b^	0^b^
Vitamin A	8.82	9.21^a^	8.57^b^	8.37^b^
Vitamin C	0	0^b^	0^a^	0^a^
MPA	23.60	26.32^a^	22.19^b^	21.73^b^

*Note:* The values are presented as the median. Superscript letters indicate results of pairwise comparisons following the Kruskal–Wallis test. Groups sharing the same letter (e.g., aa or bb) are not significantly different (*p* > 0.05), whereas groups with different letters (ab) differ significantly (*p* < 0.001).

Abbreviations: MPA, mean probability of adequacy; NPNL, non‐pregnant and non‐lactating; PLW, pregnant and lactating.

## 4 Discussion

The FBDG of Bangladesh encourages the intake of recommended levels of cereals, fruits, vegetables, legumes, animal‐source foods, nuts and seeds, and milk and milk products while limiting the intake of sugar, sugar‐sweetened beverages, oils, oily foods, and salt. In addition, the guidelines recommend age‐ and sex‐specific intake levels of energy, macronutrients, and micronutrients following the ICMR. However, the adherence of food consumption to recommended dietary guidelines for the adult population in Bangladesh has never been studied before. Thus, the present study explored the dietary intake of different food groups and nutrients among the adult men and women of Bangladesh. It also examined the adherence of the intake of different foods and nutrients to the daily dietary recommendations level of the FBDG of Bangladesh.

The present study has assessed the deviation of food consumption from the recommended dietary guidelines set for the Bangladeshi adult population. The results documented a substantial shortfall in the proportion of adults following dietary recommendations for all food groups, particularly meeting dietary recommendations for fruits, followed by milk and milk products, non‐leafy vegetables, and leafy vegetables. Less than 10% of the population appeared to eat the recommended amount of those food groups. Moreover, fish, meat, and egg intake were below the recommended level for more than half of the participants. Among the consumed food groups, cereals and grains dominated their daily dietary energy contribution (over 70% of daily energy), while the percent contribution of protein sources, fruits, vegetables, nuts, seeds, and so forth was low. Previous studies also identified an inadequately diverse diet among adult men and reproductive‐aged women (Akter et al. 2021; Islam, Jubayer, et al. 2023; Islam et al. 2024; Sinharoy et al. 2018). Their diet is predominantly cereal and grain‐based with inadequate intake of milk, fruits, leafy vegetables, fish, meat, egg, nuts, seeds, and so forth. A similar inadequately diverse diet dominated by cereal‐based products was also mentioned among pregnant and lactating mothers by several studies in Bangladesh (Islam et al. 2022; Shamim et al. 2016). Several underlying factors, including the high price of animal‐sourced foods, fruits, and so forth, low affordability, food insecurity, and lack of awareness, contributed to inadequate intake of different food items and led consumers to cereal‐based diets (Islam, Nowar, et al. 2023; Khatun et al. 2020). According to previous studies, 43% of the population in Bangladesh cannot afford a recommended diet due to high costs and food insecurity (Islam, Nowar, et al. 2023). This cereal‐based diet devoid of milk and milk products, fruits, leafy and non‐leafy vegetables, and so forth, might make them vulnerable to micronutrient deficiencies.

In the current study, most of the participants' intake of sugar was within the maximum allowable intake level (≤ 25 g/day), but only half of them met the recommendation regarding salt intake (< 5 g/day). Previous studies and national surveys in Bangladesh also identified a higher intake of salt beyond the recommended level by the adult population (NIPSOM et al. 2020; Zaman et al. 2017). Their findings showed that the mean salt intake was 9.0–17 g daily. Added salt intake during meals, salty sauces, salty and spicy fast foods, salty processed foods, salty ready‐to‐eat or street foods, and so forth are several causes of higher salt intake in Bangladesh. According to the STEPS survey, about 45% of men and 51.5% of women were used to always or often adding salt to their food before eating or as they were eating (NIPSOM et al. 2020). Another study by the National Heart Foundation of Bangladesh also reported that 69% of the adult population (94% in rural and 44% in urban areas) were habituated to take extra salt during their meals (Zaman et al. 2017). High salt intake is a well‐recognized factor related to hypertension, cardiovascular diseases, and other non‐communicable diseases (He and MacGregor 2007).

The findings of our study are comparable to the findings from previous studies in other countries, including neighboring countries of Bangladesh. A study in India found that women consistently consumed fewer food groups than other household members, with significant gaps in non‐staple foods such as vitamin A‐rich fruits and vegetables, meat, and dairy (Gupta et al. 2020). Evidence from Qatar, The Gambia, Germany, Egypt, and a global review reveals widespread non‐adherence to dietary guidelines across both high‐ and low‐/middle‐income countries (Ali et al. 2022; Ansari and Samara 2018; Islam et al. 2024; Leme et al. 2021; Stroebele‐benschop et al. 2018; Thani et al. 2018). Around 40% of populations fail to meet key recommendations, with common issues including low intake of fruits, vegetables, whole grains, and legumes, and high intake of refined grains, added sugars, and animal‐source foods. In Qatar, over 83% of adults fell short on key food groups, with poor adherence linked to obesity and metabolic risks (Thani et al. 2018). Gambian diets were high in refined grains and sugars, but low in fruits and vegetables (Ali et al. 2022). In Germany, most university students missed dietary targets, though habit strength influenced food choices (Stroebele‐benschop et al. 2018). In Egypt, adherence was under 45% for most groups, with notable gender gaps and links to the perceived importance of healthy eating (Ansari and Samara 2018).

According to the findings of the present study, most of the participants (96.2%) consumed the recommended amount of protein (7.5%–20% of total energy), while the fat intake was below the recommended range (< 20% of total energy) by a higher percentage (65.5%). The ICMR recommends taking at least 20% of daily energy from fat for adults and pregnant and lactating women (ICMR‐NIN 2024). However, in the current study, only 31.3% of the men and 29% of the women had met the recommended level of fat intake (20%–30% of total energy). Although the fat and oil intake has increased in Bangladesh during the last decades, a higher percentage of the population still consumes inadequate dietary energy from fat (BBS (Bangladesh Bureau of Statistics) 2022; Islam et al. 2022). Despite a higher proportion of adult men and women meeting the recommended level of protein, the quality of the protein could be an issue of concern. In the present study, more than half of the protein was contributed by cereals, roots, and tubers, while less than one‐third of the protein came from animal source foods. On the contrary, around 90% of the study participants exceeded the recommended level of carbohydrate. A cereal‐dominated diet contributed a higher intake of carbohydrate among Bangladeshi adults.

Moreover, a lower proportion of the participants met the adequacy of riboflavin, calcium, vitamin A, zinc, vitamin B_12_, iron, vitamin C, vitamin B_6_, and magnesium intake. The overall mean probability of adequacy (MPA) was also low (23.6%). This inadequate intake of different micronutrients was also highlighted by several previous studies in Bangladesh among adult men, women of reproductive age, and pregnant and lactating women (Akter et al. 2021; Islam et al. 2021, 2024; Islam, Nowar, et al. 2023). Inadequate intake of milk and milk products and other animal‐source foods (ASF) inadequately supplies calcium, iron, and vitamin B_12_ for them. The cereal‐based diet devoid of milk and milk products, fruits, leafy and non‐leafy vegetables, meat, fish, egg, and so forth, mentioned above might contribute to inadequate intake of those multiple essential micronutrients. Thus, FBDG recommends an adequately diverse and balanced diet to ensure adequate intake of important vitamins and minerals.

Sex differences in food consumption were also observed in the study findings. Adult men showed better adherence to non‐leafy vegetables, fruits, meat, fish, eggs, pulses, and legumes, whereas women were likely to adhere to the recommended intake of salt and fats. However, there was no significant difference in energy and macronutrient intake among the men, NPNL, and PLW. In the case of micronutrients, males had a higher probability of adequacy for calcium, iron, thiamin, riboflavin, vitamin A, and overall MPA compared to females. A previous study also highlighted a higher intake of dietary energy and higher diet quality among males than females in Bangladesh (Sudo et al. 2004). As mentioned earlier, women in India consistently consumed fewer food groups than other household members, with significant gaps in non‐staple foods such as vitamin A‐rich fruits and vegetables, meat, and dairy (Gupta et al. 2020). Moreover, in South Asia, including Bangladesh, women faced discrimination and were deprived of their adequate level of intake, especially in the case of the distribution of nutrient‐dense, high‐quality food (Harris‐Fry et al. 2017). Such intra‐household food allocation might make women more vulnerable to inadequate dietary intake compared to males in the same family.

## 5 Strengths and Limitations of the Study

The study has some significant strengths and a few limitations. To the best of our knowledge, this is the first study to examine the adherence of the intake of different foods and nutrients to the daily dietary recommendations level of the FBDG of Bangladesh. Moreover, the NSB survey collected individual‐level intake of the household members using the food weight record method. The survey was repeated in 10% of the houses to ensure data validity. No data was collected throughout the fasting month of Ramadan and 2 weeks after the day of the Muslim holy festival of live‐stock sacrifice. This provides less estimation error in the dietary intake of the study participants. The use of the comprehensive Bangladeshi Food Composition Table/Database for estimating the nutrient content of the consumed food items by adult men and women gives more reliability. Furthermore, the nutrient intake adequacy was assessed by comparing the observed intake with the age‐, sex, physiological state‐specific AMDR and EAR of the ICMR.

This study has several limitations that should be considered when interpreting the findings. First, the analysis was based on secondary data from a nationally representative dietary survey. We had no control over measurement procedures or access to the full sampling design details. Although the survey followed a stratified cluster sampling design, the dataset did not include sampling weights, cluster, and strata, limiting our ability to produce nationally weighted estimates and survey design‐adjusted statistical analyses. Second, the estimation of usual nutrient intake relied on repeat dietary data from only 10% of the sample, which may be insufficient to fully capture intra‐individual variability. Nonetheless, we applied statistical methods (probability approach) to estimate usual intake and assess nutrient adequacy. Fourth, no seasonal adjustment was made in the analysis, although data were collected across an entire year. Seasonal changes in food availability may influence dietary patterns, and we acknowledge this as a limitation. Similarly, although the weighed food record method is considered accurate, it may still be affected by social desirability bias, as participants could have altered their intake during the survey period. Fifth, we did not perform sensitivity analyses, such as using alternative cut‐offs or food composition databases. The dataset used the Bangladesh Food Composition Table, which is the most appropriate for this population. However, we recognize that such analyses could help assess the robustness of the results and recommend this for future studies. Lastly, while we discussed individual‐level dietary adherence, we acknowledge that structural barriers such as food affordability, availability, and cultural preferences play a critical role in shaping food choices. These factors were not captured in the current dataset and should be explored in future research.

## 6 Implications of the Study

Since the food‐based dietary guidelines help the population reach the nutrient recommendations and guide national food and nutrition policies and the food industry, sustainable approaches are required to promote healthy eating habits, which must consider existing nutritional problems and traditional diets. Thus, consequent revision in FBDG would affect the diet of the Bangladeshi population to improve their nutritional status. Furthermore, a notable shift in the current food supply chain and consumption patterns is required to maximize adherence to country‐specific FBDG. The findings of this study have important implications for nutrition policy, dietary interventions, and public health planning in Bangladesh. The low adherence to the national FBDG, especially in the consumption of dairy, fruits, vegetables, and pulses, highlights a significant gap between recommended and actual dietary patterns among Bangladeshi adults. This dietary imbalance contributes to widespread inadequacy in essential micronutrients such as calcium, vitamin A, riboflavin, and iron—nutrients critical for overall health and disease prevention. The heavy reliance on cereals and grains for energy, with limited intake of nutrient‐dense foods, suggests that national strategies should prioritize dietary diversification. Increasing access to and consumption of under‐utilized food groups is essential to improving micronutrient intake. Moreover, the lower nutrient adequacy observed among women points to the need for gender‐sensitive nutrition programs that address women's specific dietary needs. The study findings underscore the importance of strengthening nutrition education, improving food system access to affordable and diverse foods, and integrating FBDG adherence into national health promotion strategies. Policymakers and program implementers should consider these gaps when designing targeted interventions to improve dietary quality and reduce micronutrient deficiencies across the population.

## 7 Conclusion

The study highlighted a lower adherence proportion for fruits, followed by milk and milk products, non‐leafy vegetables, leafy vegetables, and pulse and legumes. Similarly, more than half of the participants did not adhere to the dietary recommendations regarding fish, meat, and egg intake. Cereals dominated the diet, with around 90% exceeding the upper level of recommendation. Women had higher adherence to cereals, while men adhered more to fruits, animal‐source foods, and milk. Almost all the participants met the protein intake guideline, while most participants consumed fat below and carbohydrate above the recommended range. Several micronutrient inadequacies were widespread, especially for calcium, riboflavin, vitamin B12, and vitamin C. Men had better iron and thiamin adequacy, whereas women, particularly PLW, showed higher adequacy for magnesium, zinc, folate, and vitamin B6. MPA was highest in men, followed by NPNL and PLW. The intake of milk and milk products, fruits, vegetables, pulses, and animal‐based foods could be increased to meet the dietary recommendations of these food groups and the adequacy of different micronutrients. Thus, to ensure the nutritional well‐being of the people of Bangladesh, a food system approach could be implemented to make the mentioned food groups available and affordable to all people and make them aware of consuming those food groups in adequate amounts.

## Author Contributions

**Md. Hafizul Islam:** conceptualization (equal), data curation (equal), formal analysis (lead), methodology (equal), software (lead), writing – original draft (lead), writing – review and editing (equal). **Oumma Halima:** conceptualization (equal), data curation (equal), formal analysis (supporting), methodology (supporting), supervision (supporting), writing – original draft (supporting), writing – review and editing (equal). **Nazma Shaheen:** conceptualization (equal), formal analysis (supporting), methodology (equal), supervision (lead), writing – original draft (supporting), writing – review and editing (equal).

## Ethics Statement

The present study is a secondary analysis of the consumption data of the Nutrition Survey of Bangladesh (NSB) 2017–18, which is publicly available. The NSB obtained ethical approval from the Faculty of Biological Science, University of Dhaka. Written informed consent was obtained from the study participants during the administration of the field survey. Individuals were informed that participation was voluntary and that they could withdraw at any point in the study process. No personal identifier was collected, and the confidentiality of the information was maintained.

## Conflicts of Interest

The authors declare no conflicts of interest.

## Supporting information


**Table S1:** fsn371056‐sup‐0001‐TableS1.docx.

## Data Availability

The dataset supporting the conclusions of this article is available on the website of FAO/WHO GIFT (Global Individual Food Consumption Data Tool): https://www.fao.org/gift‐individual‐food‐consumption/data/en.
