# Supplementary material for: Food Consumption Among the Adult Men and Women of Bangladesh and Its Adherence to National Food‐Based Dietary Guidelines
Source: Food Sci Nutr. 2025 Oct 8;13(10):e71056. doi: 10.1002/fsn3.71056 (PMC12507732; doi:10.1002/fsn3.71056)
Supplement: Supplementary file 1 — Table S1: fsn371056‐sup‐0001‐TableS1.docx. [file FSN3-13-e71056-s001.docx]

Supplementary Table 1: Socio-demographic characteristics of the study participants

| **Socio-demographic characteristics** | **n (%)** |
| --- | --- |
| Age (years) (1551)  Mean (SD)  19-30  31-40  41-50 | 30.91 (7.88)  897 (57.8)  441 (28.4)  213 (13.7) |
| Sex (1551)  Men  Women | 758 (48.9)  793 (51.1) |
| Region (1551)  Rural  Urban | 997 (64.3)  554 (35.7) |
| Educational level (1551)  No formal education  Primary  Secondary  Higher secondary and above | 310 (20.0)  467 (30.1)  579 (37.3)  195 (12.6) |
| Household size (1551)  1-4 members  More than 4 members | 1107 (71.4)  444 (28.6) |
| Physiological status of the women (793)  Non-pregnant and non-lactating  Pregnant and lactating | 464 (58.5)  329 (41.5) |
